# Nerve root metastasis of gastric adenocarcinoma: A case report and review of the literature

**DOI:** 10.1016/j.ijscr.2019.07.001

**Published:** 2019-07-08

**Authors:** Alessandra Di Sibio, Lucia Romano, Antonio Giuliani, Marco Varrassi, Maria Carmela De Donato, Antonio Iacopino, Marco Perri, Mario Schietroma, Francesco Carlei, Ernesto Di Cesare, Carlo Masciocchi

**Affiliations:** a Department of Radiology, S. Salvatore Hospital, Via L. Natali, 1, 67100, L’Aquila, Italy; b Department of Surgery, University of L’Aquila, L’Aquila, Italy; c Department of Applied Clinical Science and Biotechnology, University of L’Aquila, L’Aquila, Italy

**Keywords:** Metastatic gastric cancer, Spinal nerve root ganglion neoplasm, Metastasis, Radiation therapy

## Abstract

**Introduction:**

Nerve root metastasis without extension from an adjacent process has been seldom reported in case of solid tumors. We describe a case of solitary nerve root metastasis of gastric adenocarcinoma, likely due to hematogenous spread.

**Case presentation:**

A 75-year-old man presented with radiculopathy refractory to medical treatment. MRI and CT demonstrated a right-sided S1 nerve root mass involving the spinal ganglion in its intra-foraminal region with avid enhancement, initial erosive bone changes on sacral foramina and focal hyperaccumulation on 18F – FDG CT-PET, suspicious for metastasis. The histopathological examination confirmed a metastasis of gastric adenocarcinoma.

**Discussion:**

A review of the current literature revealed only ten cases of hematogenous metastases to spinal nerve root ganglia; the primary lesions in those cases were an oat cell carcinoma of the lung, two cases of colonic adenocarcinoma, a case of uterine adenocarcinoma, a ductal breast carcinoma, a Ewing’s sarcoma, a Renal Cell Carcinoma, a gastro-intestinal stromal tumor, a follicular thyroid carcinoma, a pulmonary adenocarcinoma.

**Conclusion:**

In the setting of a known malignancy, a nerve root metastasis should be considered in the differential diagnosis of a nerve root mass, although it occurs very rarely.

## Highlights

• Metastatic involvement of peripheral nerves by perineural invasion from an adjacent cancer is not uncommon.
• Nerve root metastasis without extension from an adjacent process has been seldom reported in case of solid tumors.
• No cases of gastric adenocarcinoma metastasis to the nerves have been reported to date.
• In case of known malignancy and refractory pain, the possibility of a nerve root metastasis should be considered.

## 1 Introduction

Nerve root metastases of solid tumors have been rarely reported and, to the best of our knowledge, no cases of gastric adenocarcinoma metastasis to the nerves have been reported to date [1,2]. Nerve root metastases can mimic clinically a radiculopathy. Radiological findings of nerve root metastases might mimic those of peripheral nerve sheath tumors (PNST) [3], which represent the principal differential diagnosis. We describe a case of a patient presenting a S1 nerve root metastasis of gastric adenocarcinoma. Our work is in line with the SCARE criteria [4].

## 2 Case presentation

On May 2018, a 75-year-old man was referred to our Department due to an increasing low-back pain and right-sided radicular pain irradiating down to the right lower limb, along the posterior thigh and the postero-lateral aspect of the lower extremity up to the lateral aspect of the foot, numbness in the sole of his right foot, especially marked on the plantar surface of his toe (VAS 8); the patient’s pain was refractory to conventional medical treatment. He complained also with a subjective decrease in strength in his right lower limb; his walk was slow and difficult due to the pain.

On clinical examination hypoesthesia at the right S1 and S2 dermatome areas and moderate muscle weakness in the right lower limb (3–4/5) were observed, reflexes were bilaterally normally elicitable and plantar response was flexor.

**Fig. 1:**
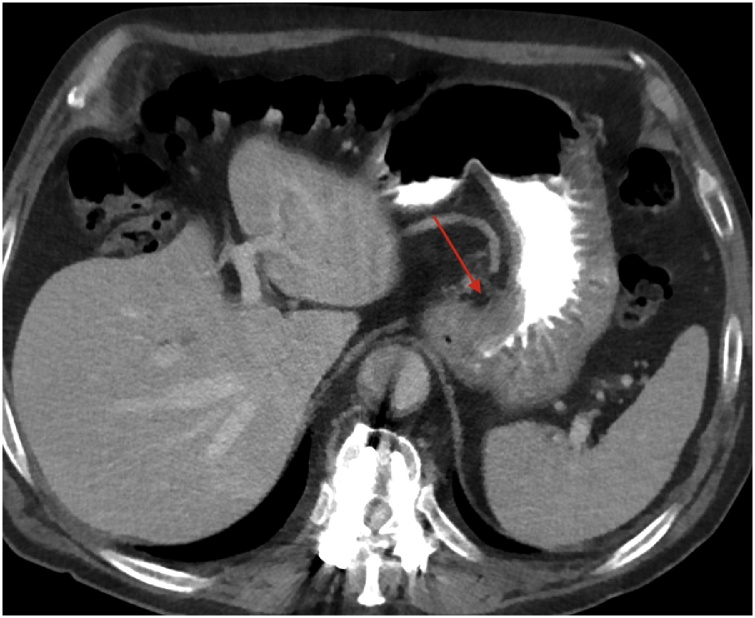
Axial CT venous phase (January 2016) demonstrated the irregular wall thickening of the subcardial lesser curvature (arrow) referable to the primary gastric neoplasm.

His past medical history included total gastrectomy, omentectomy, regional lymphadenectomy (D2) and reconstruction by mean Roux-en-Y anastomosis 2 years before (on January 2016) due to a gastric adenocarcinoma in the subcardial region; the histopathological diagnosis was poorly-differentiated gastric adenocarcinoma of intestinal type and 4/14 lymphnodes were positive for metastatic disease (pT3 pN2 R0; stage IIIa). Before surgery (January 2016), he underwent a whole body CT scan using a first-generation 640-slice CT scanner (Aquilion One, Toshiba Medical Systems, Outerwear, Japan), that showed the subcardial neoplasm measuring about 25 × 15 mm (Fig. 1) without sign of distant metastases. The patient had a family history of gastric adenocarcinoma, his mother being affected. After surgery, the patient underwent 4 courses of adjuvant chemotherapy consisting of Oxaliplatin 120 mg/m^2^ and Capecitabine 825 mg\mq^2^. In clinical and radiological follow-up, the last on December 2017 (Fig. 3a), no local recurrence or metastases were observed.

**Fig. 2:**
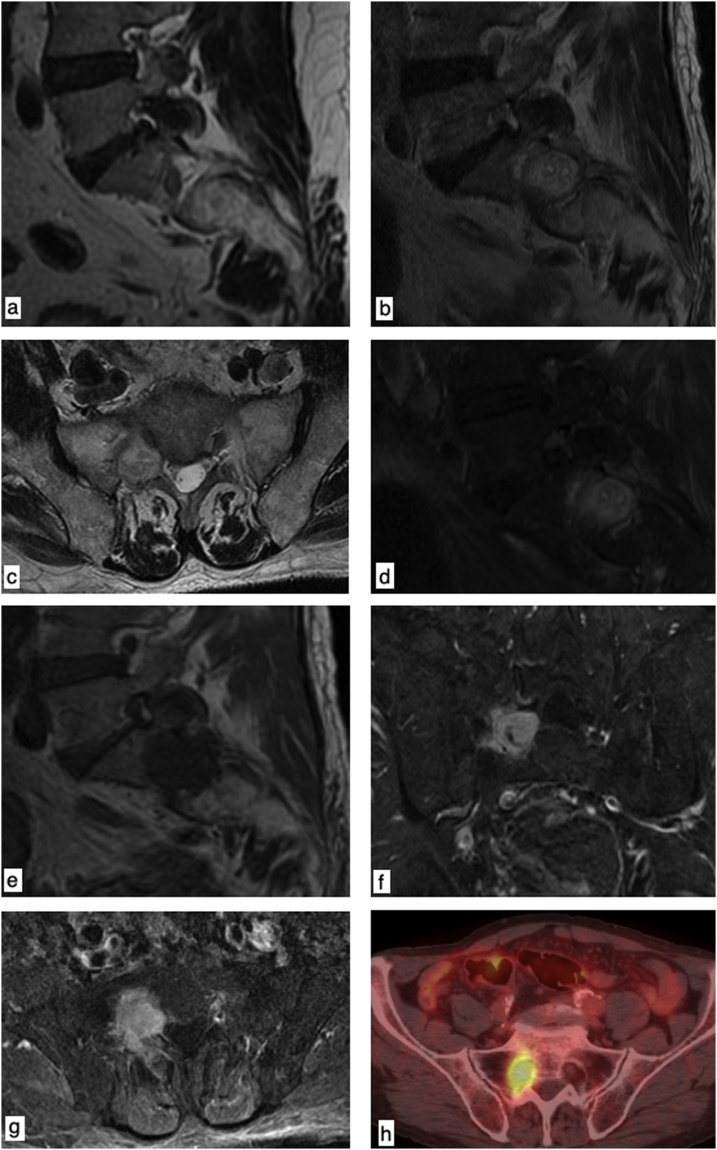
Sagittal TSE T2-weighted image (July 2015) showing no abnormal findings in the L5-S1 foraminal space (a). Sagittal TSE T2-weighted image (b), axial TSE T2-weighted image (c) and sagittal fat-sat TSE T2-weighted image (d) showing a hyperintense round-shaped lesion involving the right L5-S1 foraminal space. Sagittal SE T1-weighted image (e) showing an hypointense mass in the L5-S1 foraminal space; coronal (f) and axial (g) fat-sat SE T1-weighted image after intravenous contrast administration showing avid enhancement of the lesion. 18F – FDG (18F-Fluorodeoxyglucose) CT-PET (Fig. 2h) showing focal hyperaccumulation within the sacral foramina of right S1 nerve root (SUV max. 5.2).

His past medical history also included a L1 vertebral body fracture 3 years before (on July 2015), requiring a lumbar MRI (Magnetic Resonance Imaging) performed on a 1.5T General Electric Medical System (Fig. 2a), with subsequent surgical stabilization.

**Fig. 3:**
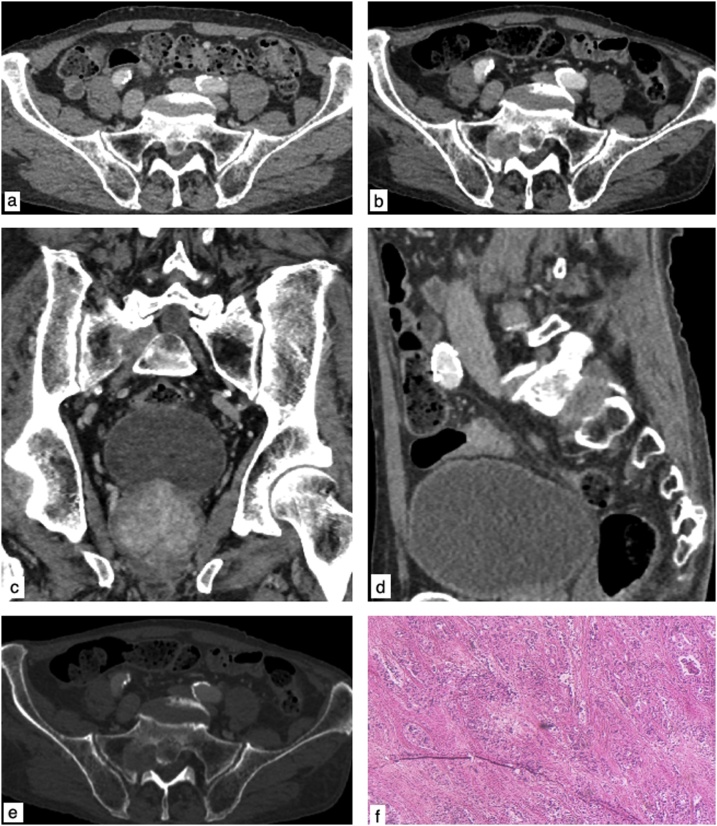
Axial CT venous phase (December 2017) showed no abnormal findings in the L5-S1 foraminal space (a). Axial CT venous phase (b), with coronal (c) and sagittal (d) reconstructions, showing the slightly hypervascular right-sided S1 nerve root mass. Axial CT bone window (e) showing initial erosive bone changes on right S1 sacral foramina. Histopathological examination stained with hematoxylin eosin (f) confirmed a metastasis of gastric adenocarcinoma.

On May 2018, patient underwent a lumbar MRI using a 1.5T General Electric Medical System, that showed a significant enlargement of the intraforaminal right S1 nerve root due to the presence of a right-sided S1 nerve root mass involving the spinal ganglion in its intra-foraminal region and growing along the spinal nerve root, measuring about 25 × 20 × 25 mm; the mass showed homogeneously hypo-intensity signal on SE T1-weighted images (repetition time [TR] 400 ms; echo time [TE] 14 ms; Fig. 2e), significant hyper-intense signal on TSE T2-weighted images (repetition time [TR] 3960 ms; echo time [TE] 114 ms; Fig. 2b–d), avid and homogeneous contrast enhancement on SE T1-weighted images (repetition time [TR] 400 ms; echo time [TE] 14 ms; Fig. 2f, g) enhanced by gadolinium-diethylenetriaminepenta-acetic acid (16 ml Dotarem, 0.2 ml/kg). The mass was not evident in the previous MRI (Fig. 2a) – CT (Fig. 3a) exams and was considerate suspicious for metastasis.

Afterwards he received an 18F – FDG (18F-Fluorodeoxyglucose) CT-PET (Fig. 2h) that showed an area of focal hyperaccumulation within the sacral foramina of right S1 nerve root (SUV max. 5.2), suggestive of metastasis.

He also underwent a whole body CT scan using a first-generation 640-slice CT scanner (Aquilion One, Toshiba Medical Systems, Outerwear, Japan), that confirmed the presence of the slightly hypervascular right-sided S1 nerve root mass (Fig. 3b–d) and showed initial erosive bone changes (Fig. 3e) on S1 sacral foramina; no other CT alterations suspicious for metastatic lesion was detected in the brain, thorax, abdomen and pelvis.

A fine-needle aspiration biopsy (FNAB) was performed and the histologic examination confirmed a metastasis of gastric adenocarcinoma (Fig. 3f).

Following interdisciplinary discussion, the patient was referred for radiotherapy; radiation to the right S1 nerve root mass was performed to relieve the local pain and a total dose of 16 Gy in two fractions was given. The patient reported a reduction of pain intensity (VAS 6) and muscle weakness in the right lower limb (2+/5).

## 3 Discussion

Although metastatic involvement of peripheral nerves by perineural invasion from an adjacent cancer is not uncommon, especially in the context of head and neck tumors [5], nerve root metastasis without extension from an adjacent process has been seldom reported in case of solid tumors [1,2] and no cases of gastric adenocarcinoma metastasis to the nerves have been reported to date.

Stomach cancer is the fifth most frequently diagnosed cancer and the third leading cause of cancer death, is responsible for over 1.000.000 new cases in 2018 and an estimated 783.000 deaths (equating to 1 in every 12 deaths globally) [6]. Gastrectomy with regional lymphadenectomy (D2) is the curative-intent surgical treatment for all patients with gastric adenocarcinoma that fit to undergo surgery [[7], [8], [9], [10], [11]], as carried out on our patient. In patients with stage III gastric cancer, as our patient, multiple meta-analyses showed that post-operative adjuvant chemotherapy is associate with a survival benefit, even if the indications and characteristics of this therapy for locally advanced gastric cancer are still under investigation [12,13].

We report the case of a patient with S1 nerve root metastasis of gastric adenocarcinoma. To our knowledge, only ten cases of metastases to spinal nerve root ganglia, arising from distant solid tumors, have been reported in the literature, our case is the eleventh reported case of spinal nerve root metastasis [2] and the first reported case of metastasis to a sacral nerve root from gastric adenocarcinoma.

Various routes of dissemination have been proposed, which include direct invasion (i.e., Pancoast tumors), lymphohematogenous (breast, lung, head and neck tumors), spinal fluid and retrograde endoneurial spread [2]. Our case was likely due to hematogenous spread.

A review of the current literature revealed only ten cases of hematogenous metastases to spinal nerve root ganglia; the primary lesions in those cases were an oat cell carcinoma of the lung, two cases of colonic adenocarcinoma, a case of uterine adenocarcinoma, a ductal breast carcinoma, a Ewing’s sarcoma, a RCC (Renal Cell Carcinoma) [3,14], a gastro-intestinal stromal tumor (GIST) [[15], [16], [17]], a follicular thyroid carcinoma [2], a pulmonary adenocarcinoma [18].

Clinically, nerve root metastases mimic radiculopathy and/or cauda equina syndrome, with the earliest symptoms being pain and weakness, followed by sensory loss, and bowel and bladder dysfunction [2]. According to Jung et al., increasing radicular symptoms is the presenting symptom in 90–95% of patients [2], which was manifest in our patient, who first developed low-back pain and right-sided radicular pain.

Nerve root metastases manifest on CT/MRI as thickening of involved nerve root with contrast enhancement and, sometimes, erosive changes in the adjacent bones [2,3,14,15,18], which was also observed in our case.

The radiological appearance of nerve root metastasis might mimic that of a peripheral nerve sheath tumor (PNST) [3], which represent the main radiological differential diagnosis.

Tumors of the peripheral nerves are relatively common; these tumors arise from either the neuronal tissue or from the cells of the neural sheath [3]. PNST can be further subdivided in benign, accounting for more than 90%, mostly represented by schwannomas or neurofibromas, and malignant lesions (MPNST) [19,20]. MPNST are rare tumors, mainly diagnosed in patients with history of Neurofibromatosis type 1 or in previously radiated patients, aggressive and with poor prognosis [14,19].

Radiological appearance of MPNST is non - specific, however these lesions tend to be isointense to muscle in T1 weighted-sequences, while in our case the metastatic lesion was hypointense to muscle in T1 weighted-sequence. Both MPNST and metastases show hyperintensity on T2 weighted-sequences, avid contrast enhancement with increased metabolic activity on F-18 FDG-CT-PET [[19], [20], [21]].

Although differential diagnosis among MPNST and benign PNST is often challenging, some MRI features can be helpful. Differently from benign PNST, MPNST usually tend to be larger, to show less-defined margins and a more inhomogeneous contrast enhancement [19,20].

Presence of cystic or hemorrhagic areas within the lesion and perilesional edema are more typical of malignant lesion; moreover benignant lesion are rarely larger than 5 cm and characterized by a more homogeneous signal on MRI sequences [[19], [20], [21]].

A central hypointense region on T2-weighted images (target sign) is typical in neurofibromas, that show often focal central enhancement while widespread and-or peripheral enhancement is more typical of malignant neoplasms [[19], [20], [21]].

Lastly, bone involvement, often with an erosive pattern, is highly suspicious for malignancy [[18], [19], [20]], as confirmed in our case.

## 4 Conclusion

Our case shows that, in the setting of a known malignancy, the possibility of a nerve root metastasis should be considered in patients with history of gastric cancer and increasing radicular symptoms, especially in case of refractory pain.

## Conflicts of interest

No conflict of interest.

## Sources of funding

No source of funding.

## Ethical approval

This study was approved by the Research Ethics Committee of the University of L’Aquila.

## Consent

The authors obtained patient consent to use all the images presented.

## Author’s contribution

Alessandra Di Sibio, Lucia Romano, Antonio Giuliani, Marco Varrassi: Writing the paper.

Maria Carmela De Donato, Antonio Iacopino, Marco Perri: Data collection and analysis.

Mario Schietroma, Francesco Carlei, Ernesto Di Cesare, Carlo Masciocchi: Study concept.

Alessandra Di Sibio, Lucia Romano, Antonio Giuliani, Marco Varrassi, Maria Carmela De Donato, Antonio Iacopino, Marco Perri, Mario Schietroma, Francesco Carlei, Ernesto Di Cesare, Carlo Masciocchi: Critical revision.

## Registration of research studies

N/A.

## Guarantor

Prof. Ernesto Di Cesare.

## Provenance and peer review

Not commissioned, externally peer-reviewed.

## References

[1] Li L., Wu Y., Hu L., Xu H., He H., Hu D. (2016). Metastatic nerve root tumor: a case report and literature review. Mol. Clin. Oncol..

[2] Keen J., Milosavljevic E., Hanna G., Gospodarev V., Raghavan R., Ghostine S. (2016). Rare intradural cervical nerve root metastasis of follicular thyroid carcinoma. Cureus.

[3] Schulz M., Lamont D., Muthu T., Hussain Z., Balakrishnan V. (2009). Metastasis of breast cancer to a lumbar spinal nerve root ganglion. Spine (Phila Pa 1976).

[4] Agha R.A., Borrelli M.R., Farwana R., Koshy K., Fowler A., Orgill D.P., For the SCARE Group (2018). The SCARE 2018 statement: updating consensus Surgical CAse REport (SCARE) guidelines. Int. J. Surg..

[5] Uchida K., Kobayashi S., Yayama T., Muramatsu J., Kurokawa T., Imamura Y., Baba H. (2008). Metastatic involvement of sacral nerve roots from uterine carcinoma: a case report. Spine J..

[6] Bray F., Ferlay J., Soerjomataram I., Siegel R.L., Torre L.A., Jemal A. (2018). Global cancer statistics 2018: GLOBOCAN estimates of incidence and mortality worldwide for 36 cancers in 185 countries. CA Cancer J. Clin..

[7] Wohnrath D.R., Araujo R.L.C. (2019). D2 lymphadenectomy for gastric cancer as an independent prognostic factor of 10-year overall survival. Eur. J. Surg. Oncol..

[8] Schietroma M., Cecilia E.M., Carlei F., Sista F., De Santis G., Piccione F., Amicucci G. (2013). Prevention of anastomotic leakage after total gastrectomy with perioperative supplemental oxygen administration: a prospective randomized, double-blind, controlled, single-center trial. Ann. Surg. Oncol..

[9] Popivanov G., Tabakov M., Mantese G., Cirocchi R., Piccinini I., D’Andrea V., Covarelli P., Boselli C., Barberini F., Tabola R., Ursi P., Cavaliere D. (2018). Surgical treatment of gastrointestinal stromal tumors of the duodenum: a literature review. Transl. Gastroenterol. Hepatol..

[10] Giuliani A., Romano L., Papale E. (2019). Complications post-laparoscopic sleeve gastric resection: review of surgical technique. Minerva Chir..

[11] De Manzoni G., Marrelli D., Baiocchi G.L. (2017). The Italian Research Group for Gastric Cancer (GIRCG) guidelines for gastric cancer staging and treatment: 2015. Gastric Cancer.

[12] Jang S.H., Jung Y.J., Kim M.G., Kwon S.J. (2018). The prognostic significance of compliance with postoperative adjuvant chemotherapy in patients with stage III gastric cancer: an observational study. J. Gastric Cancer.

[13] Goetze O.T., Al-Batran S.-E., Chevallay M., Monig S.P. (2018). Multimodal treatment in locally advanced gastric cancer. Updates Surg..

[14] Cabrilo I., Burkhardt K., Schaller K., Tessitore E. (2013). Renal carcinoma relapse presenting as a peripheral nerve sheath tumor: a case report and brief review of the literature. Neurochirurgie.

[15] Akiyama K., Numaga J., Kagaya F., Takazawa Y., Suzuki S., Koseki N., Kato S., Kaburaki T., Kawashima H. (2004). Case of optic nerve involvement in metastasis of a gastrointestinal stromal tumor. Jpn. J. Ophthalmol..

[16] Sista F., Abruzzese V., Schietroma M., Amicucci G. (2013). Concomitant gastrointestinal stromal tumor of the stomach and gastric adenocarcinoma in a patient with Billroth 2 resection. Case Rep. Surg..

[17] Sista F., Pessia B., Abruzzese V., Cecilia E.M., Schietroma M., Amicucci G. (2015). Twelve years of gastric GIST. A retrospective study of laparoscopic and open approach. Ann. Ital. Chir..

[18] Slotty P.J., Cornelius J.F., Schneiderhan T.M., Alexander K.M., Bostelmann R. (2013). Pulmonary adenocarcinoma metastasis to a dorsal root ganglion: a case report and review of the literature. J. Med. Case Rep..

[19] Aran S., Duran G.S., Potigailo V., Kim A.E. (2017). Radiologic manifestation of the malignant peripheral nerve sheet tumor involving the brachial plexus. Radiol. Case Rep..

[20] Kakkar C., Shetty C.M., Koteshwara P., Bajpal S. (2015). Telltale signs of peripheral neurogenic tumors on magnetic resonance imaging. Indian J. Radiol. Imaging.

[21] Wasa J., Nishida Y., Tsukushi S., Shido Y., Sugiura H., Nakashima H., Ishiguro N. (2010). MRI features in the differentiation of malignant peripheral nerve sheath tumors and neurofibromas. AJR Am. J. Roentgenol..

